# Extent of peritoneal metastases from colorectal cancer is not associated with changes in thrombin generation or fibrinolysis

**DOI:** 10.1515/pp-2024-0009

**Published:** 2024-11-06

**Authors:** Mikkel Lundbech, Andreas Engel Krag, Lene Hjerrild Iversen, Birgitte Brandsborg, Anne-Mette Hvas

**Affiliations:** Department of Clinical Biochemistry, 11297 Aarhus University Hospital, Aarhus N, Denmark; Department of Clinical Medicine, Aarhus University, Aarhus, Denmark; Department of Orthopaedics, Randers Regional Hospital, Randers, Denmark; Department of Plastic and Breast Surgery, Aarhus University Hospital, Aarhus, Denmark; Department of Surgery, Aarhus University Hospital, Aarhus, Denmark; Department of Anesthesiology and Intensive Care, Aarhus University Hospital, Aarhus, Denmark; Faculty of Health, Aarhus University, Aarhus, Denmark

**Keywords:** colorectal cancer, metastases, blood coagulation, embolisms and thrombosis, cytoreductive surgery

## Abstract

**Objectives:**

Cancer cells can activate coagulation and inhibit fibrinolysis. The aim was to investigate the association between the burden of peritoneal metastases from colorectal cancer (PM-CRC) and biomarkers reflecting thrombin generation and fibrinolysis.

**Methods:**

A cohort of 55 patients with PM-CRC scheduled for cytoreductive surgery. Patients were grouped by the peritoneal cancer index (PCI) assessed intraoperatively into limited PM-CRC (PCI≤15) and extensive PM-CRC (PCI>15). Blood samples were obtained before surgery. Thrombin generation was measured *in vivo* by thrombin-antithrombin complex (TAT) and prothrombin fragment 1+2 (F1+2), and ex vivo by the endogenous thrombin potential (ETP). Fibrinolysis was analyzed with fibrin clot lysis assay, fibrinogen, and D-dimer.

**Results:**

Non-significantly decreased thrombin generation by F1+2 (p=0.72), TAT (p=0.32), and ETP (p=0.19) were observed in patients with extensive PM-CRC (n=9) compared with limited PM-CRC (n=46). Non-significantly prolonged 50 % clot lysis time were found in patients with extensive PM-CRC than in patients with limited PM-CRC.

**Conclusions:**

Minor non-significant differences in thrombin generation and fibrinolysis were found between patients with extensive PM-CRC and limited PM-CRC. Thus, increased peritoneal metastatic burden from colorectal cancer does not seem to affect thrombin generation and fibrinolysis.

## Introduction

Cancer cells can obstruct blood flow, damage the endothelium, and release procoagulant factors [1]. Consequently, cancer patients face an increased risk of venous thromboembolism (VTE) causing increased morbidity and mortality [2, 3].

The VTE risk increases further with the presence of distant metastases, during surgical treatment, and during receipt of systemic chemotherapy [1]. A preoperative VTE prevalence of 8 % has been reported in patients with localized colorectal cancer before surgical treatment, while an incidence up to 14 % have been reported in patients with metastatic colorectal cancer within one year after treatment [4, 5].

Peritoneal metastases from colorectal cancer (PM-CRC) occur in approximately 10 % of colorectal cancer patients during their cancer trajectory reducing the 3-year overall survival to (<5–45 %) depending on the extent of PM-CRC and treatment, and other factors [6].

Selected patients with a limited extent of PM-CRC can be offered curatively intended cytoreductive surgery (CRS) with or without hyperthermic intraperitoneal chemotherapy (HIPEC) [7]. The extent of PM-CRC is intraoperatively quantified by the surgeon with, among others, the peritoneal cancer index (PCI) [8]. PCI is a strong prognostic factor of the survival following CRS with or without HIPEC [8, 9]. CRS with or without HIPEC is offered to patients with resectable and limited extent of PM-CRC such as a PCI below 15. Patients who do not fulfil these criteria are managed by surgical debulking and systemic chemotherapy [8, 10].

In addition to the increased VTE risk in cancer patients, increased coagulation has been shown to affect various pathways facilitating tumor growth and metastatic dissemination [11]. Biomarkers reflecting *in vivo* and ex vivo thrombin generation have primarily been studied in mixed cancer cohorts demonstrating an association between the presence of metastases and increased thrombin generation levels. However, whether the extent of peritoneal metastases induces changes in thrombin generation and fibrinolysis markers has been sparsely investigated 12], [13], [14], [15], [16.

Hence, we aimed to investigate the association between the extent of PM-CRC assessed by the PCI and biomarkers reflecting thrombin generation and fibrinolysis.

## Methods

The present study was a sub-study to a prospective cohort study (Changes in Coagulation in Colorectal Cancer Patients Undergoing Surgical Treatment (CONTEST), which was approved by The Central Denmark Region Committees on Health Research Ethics (journal no 1-10-72-212-20) and registered at ClinicalTrials.gov (https://classic.clinicaltrials.gov/ct2/show/NCT04744688) [17]. Written informed consent was obtained from included patients and the Helsinki Declaration was followed in all aspects.

The study design with inclusion and exclusion criteria was described at ClinicalTrials.gov [17]. In brief, from May 2021 to December 2023, patients with PM-CRC based on computed tomography scans were referred to CRS+HIPEC treatment at the Department of Surgery, Aarhus University Hospital, Denmark. A preoperative blood sample was obtained after induction of general anesthesia. The extent of PM-CRC was assessed during explorative laparotomy using the PCI by two surgeons experienced in CRS. The PCI ranges from 0 to 39 and combines the size and the extent of the peritoneal metastases in 13 abdominopelvic regions [8]. According to Danish guidelines, PM-CRC patients were treated with CRS+HIPEC if the PCI ranged from 0 to 15 and PM-CRC was present only in up to five of seven regions according to the Dutch region count score [10, 18]. If the PCI was above 15 or present in six or seven regions (Dutch region score) CRS+HIPEC was contraindicated, and the patient was excluded from the CONTEST study [10]. Furthermore, patients with PM-CRC and concomitant hepatic metastases were excluded from present study.

PM-CRC patients were divided into two groups based on their PCI. Patients with a PCI≤15 were categorized as “limited PM-CRC” and patients with PCI>15 were categorized as “extensive PM-CRC”. This PCI cut-off was regarded as the most clinically relevant threshold according to national guidelines for treatment with CRS+HIPEC for PM-CRC [10].

### Clinical data

Clinical data were obtained from electronic patient files and included age, gender, body mass index, American Society of Anesthesiologist (ASA) score, Eastern Cooperative Oncology Group (ECOG) performance score, comorbidities, VTE history, and receipt of systemic chemotherapy within three months prior to blood sampling.

All patient data were stored in a clinical database using Research Electronic Data Capture (REDCap^®^).

### Thromboprophylaxis

All included patients were administered preoperative low molecular weight heparin (Dalteparin, 5000 IU) 12 h before blood sampling. Patients receiving vitamin-K antagonists who were scored at low or moderate risk by the congestive heart failure or left ventricular dysfunction hypertension, Age≥75 (doubled), diabetes, stroke (doubled)-vascular disease, age 65–74, sex category (CHA2S2-VASc) score had their treatment paused five days before surgery. Patients scored at high risk were bridged to low molecular weight heparin three days before surgery. Direct oral anticoagulants were discontinued two days before surgery and antiplatelet therapy was discontinued minimum three days before blood sampling. Thrombo-embolic deterrent (TED) stockings were applied on the morning of the day of surgery.

### Blood sampling, preanalytical preparation, and laboratory analysis

Blood samples were drawn from an existing arterial line after induction of anesthesia into 3.2 % sodium citrate tubes (Vacuette^®^, Greiner Bio-One International GmbH, Kremsmünster, Austria). The blood samples were centrifuged at 3,000 relative centrifugal force for 25 min at room temperature within 30 min from collection. Plasma samples for *ex vivo* thrombin generation analyses were re-centrifuged at 2,500 relative centrifugal force for 15 min at room temperature before storage according to recommended guidelines [19]. All plasma samples were stored at −80 °C and until batch analyses.

*In vivo* thrombin generation was analyzed by prothrombin fragment 1+2 (F1+2) and thrombin-antithrombin complex (TAT) levels were measured using the ELISA kits F1+2 Mono and TAT micro kit (Enzygnost^®^, Siemens Healthcare GmbH; Marburg Germany). TAT levels below the detection limit at 2 ug/L were set to 1 ug/L. Analyses of F1+2 and TAT were performed in duplicate and repeated if the coefficient of variation exceeded 10 %.

Ex vivo thrombin generation was analyzed by the calibrated automated thrombogram^®^ (Thrombinoscope BV, Maastricht, the Netherlands) using 5 pM tissue factor (final concentration) and described by the endogenous thrombin potential (ETP) (nM × min) [19]. The analyses of ETP were performed in duplicate and were repeated if the coefficient of variation exceeded 10 %.

Fibrinolysis was measured with our in-house fibrin clot-lysis assay following local protocol and described by 50 % clot lysis time [20]. The clot-lysis analyses were performed in duplicate and repeated if the coefficient of variation exceeded 15 %. D-dimer and fibrinogen were conducted as standard laboratory analysis at the Department of Clinical Biochemistry, Aarhus University Hospital, Denmark, following the ISO15189- accredited routine protocol.

### Statistics

This study was an explorative sub-study to the CONTEST study which had defined the sample size [17]. QQ-plots were used to assess the distribution of continuous variables. The data was not normally distributed and therefore tested by Wilcoxen rank-sum test. Data are presented as medians with interquartile range (IQR) or (range). Correlation analysis between PCI and biomarkers was conducted using Spearman’s rank correlation.

The statistical analyses were performed in R studio (Posit Software, Boston, Massachusetts, USA) using the ‘tidyverse’ package. Data are presented in figures by GraphPad Prism 10™ (GraphPad Software, San Diego, California, USA).

## Results

In total, 55 patients with PM-CRC were included. Of these, 46 patients had limited PM-CRC (PCI≤15) and nine patients had extensive PM-CRC (PCI>15).

### Demographics

Patient demographics are presented in Table 1. Groups were comparable regarding mean age, sex, and body mass index. All patients had ECOG performance status 0 to 1 and had comparable comorbidities and administration of neoadjuvant chemotherapy.

**Table 1:** Demographics of patients with extensive extent of peritoneal metastases and limited extent of peritoneal metastases from colorectal cancer.

Variable	Extensive PM-CRC (n=9)	Limited PM-CRC (n=45)	p-Value
Age, median (IQR), years	61 (52–65)	65 (58–72)	0.14
Female, n, %	5 (56 %)	32 (70 %)	0.45
Body mass index, median (IQR), kg/m^2^	24 (22–27)	25 (23–28)	0.39

**ASA classification (I, II, III), n (%)**			0.27

I	3 (33 %)	8 (17 %)	
II	6 (67 %)	27 (59 %)	
III	0 (0 %)	11 (24 %)	

**Comorbidities**			

Charlson comorbidity index, median (IQR)	2 (1–2)	2 (1–3)	0.12
VTE history, n, %	0 (0 %)	5 (11 %)	0.58
Arterial hypertension, n, %	1 (11 %)	16 (34 %)	0.25
Arterial fibrillation, n, %	0 (0 %)	2 (4 %)	0.99
Diabetes mellitus 1+2, n, %	0 (0 %)	6 (13 %)	0.57
Receipt of neoadjuvant chemotherapy, n, %	4 (44 %)	20 (43 %)	0.99
Peritoneal cancer index, median (range)	22 17], [18], [19], [20], [21], [22], [23], [24], [25], [26], [27], [28], [29	5 1], [2], [3], [4], [5], [6], [7], [8], [9], [10], [11], [12], [13], [14], [15	0.01

Categorical variables are represented as number of patients (n) and frequencies (n/N %). Continuous variables are shown median with interquartile range (IQR) or range (min-max). Abbreviations: ASA, score, American Society of Anesthesiologists’ classification; VTE, venous thromboembolism; PM-CRC, peritoneal metastases from colorectal cancer.

### Thrombin generation and fibrinolysis

**Figure 1:**
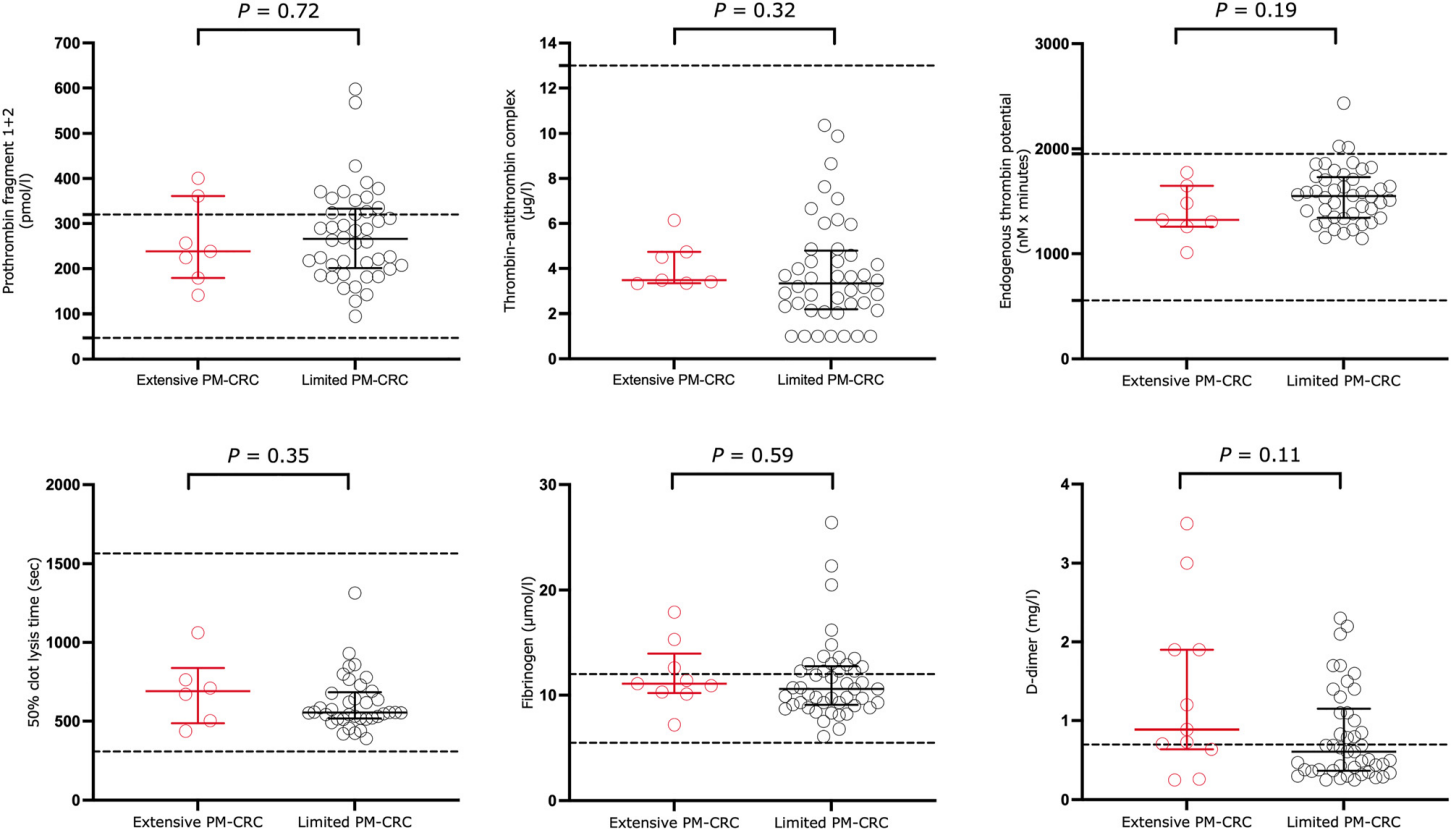
Biomarkers of thrombin generation and fibrinolysis in 55 patients with extensive (n=9) or limited (n=46) peritoneal metastases from colorectal cancer. Values are presented as median with interquartile range. Dotted lines represent reference range for in our laboratory. Abbreviations: PM-CRC, peritoneal metastases from colorectal cancer.

Thrombin generation and fibrinolysis biomarkers in patients with PM-CRC are presented in Figure 1. Patients with extensive PM-CRC had lower F1+2 (p=0.72) and lower ETP (p=0.19) than patients with limited PM-CRC with all differences being non-significant. No differences were observed in TAT (p=0.32) levels between groups. Regarding fibrinolysis, non-significantly prolonged 50 % clot lysis time (p=0.35) and increased D-dimer (p=0.11) were observed in patients with extensive PM-CRC. No differences were found in fibrinogen levels (p=0.59).

No correlation was found between extent of PM-CRC and F1+2 (Spearman rho=−0.1, p=0.96), TAT (Spearman rho=0.05, p=0.71), ETP (Spearman rho=−0.03, p=0.8), 50 % clot lysis time (Spearman rho=−0.1, p=0.5), or fibrinogen (Spearman rho=0.14, p=0.31).

There was a weak to moderate positive correlation between the extent of PM-CRC and D-dimer (Spearman rho=0.3, p=0.03).

## Discussion

The main finding of this study is that there were no significant differences in either thrombin generation reflected by F1+2, TAT, and ETP or in fibrinolysis measured by 50 % clot lysis time, fibrinogen, and D-dimer between patients with extensive PM-CRC and limited PM-CRC.

Variable risks of cancer-associated thrombosis have been reported across various cancer types with colorectal cancer being considered among cancers with a high risk for VTE [21]. The presence of metastases has also been reported to increase the risk for VTE further [22, 23]. Dickman et al. found significantly increased F1+2 levels in cancer patients with distant metastases compared with cancer patients with localized disease both with and without lymph node involvement in a mixed cohort of cancer patients [23]. Higher TAT and F1+2 levels have been observed in patients with metastatic colorectal cancer than in patients with localized colorectal cancer before treatment [24, 25]. Among these, Iversen et al. did not find an association between increased preoperative TAT and F1+2 levels and patients experiencing postoperative VTE [24]. The elevated risk of VTE in metastatic colorectal cancer may be due to a procoagulant state following overexpression of tissue factor from metastases inducing a procoagulant state [26]. Despite all patients having metastatic disease in this study, we did not observe any effect on *in vivo* and ex vivo thrombin generation levels induced by PM-CRC with all median thrombin generation markers remaining within reference range.

Our results might be explained by that PM-CRC represents limited tumor burden compared to other metastatic sites such as liver and lungs. Hence, PM-CRC might have a limited ability to release procoagulant factors into the bloodstream to induce systemic activation of thrombin generation and fibrinolysis. Furthermore, all included patients were preoperatively selected eligible for CRS having a limited overall metastatic burden solely located in the peritoneum. Hence, the overall metastatic burden could potentially be too low for the detection of markers of thrombin generation and fibrinolysis.

An association between increased D-dimer and fibrinogen levels and advanced tumor stage including tumor extension through the bowel wall and lymph node metastases has been reported previously in colorectal cancer patients 27], [28], [29], [30. Furthermore, Dai et al. observed that increased D-dimer levels were also associated with the presence of distant metastases from colorectal cancer [30]. We observed non-significantly higher D-dimer levels in patients with extensive PM-CRC than in patients with limited PM-CRC demonstrating increased fibrin turnover in patients with extensive PM-CRC. However, D-dimer is an unspecific marker of coagulation and fibrinolysis that may be elevated due increased comorbidity, VTE, cancer progression, and inflammation.

The slightly prolonged 50 % clot lysis time found in patients with extensive PM-CRC compared to those with limited CRC-PM might reflect decreased fibrinolytic capacity along with higher metastatic burden. However, 50 % clot lysis time was within the reference range for both groups.

## Strengths and limitations

The strength of the present study is that biochemical and clinical data were prospectively collected. The extent of PM-CRC was intraoperatively assessed by a few highly specialized surgeons. Both groups received the same thrombophylactic regimen. Some limitations should be considered. Only nine patients were included in the extensive PM-CRC group which might have led to a lack of statistical power and thereby risk of type II errors. We investigated patients with peritoneal metastases knowing that the extent of peritoneal metastases is difficult to assess even with the use of an international recognized assessment tool. For instance, PM-CRC and benign lesions such as fibrosis share macroscopic characteristics potentially leading to an overestimation of the PM-CRC assessed by PCI in the present as well as in other studies on peritoneal metastases [31].

## Conclusions

Minor non-significant differences in thrombin generation and fibrinolysis were found between patients with extensive PM-CRC and limited PM-CRC. Thus, increased peritoneal metastatic burden from colorectal cancer does not seem to affect thrombin generation and fibrinolysis.
